# A Rare Case of Hemoptysis: Intrapulmonary Cavitary Lesion Appearing as a Thoracic Endometriosis 

**DOI:** 10.1155/2012/351305

**Published:** 2012-08-30

**Authors:** Ali Celik, Ertan Aydın, Ulku Yazıcı, Yetkin Agackıran, Nurettin Karaoglanoglu

**Affiliations:** ^1^Department of Thoracic Surgery, Ataturk Chest Disease and Thoracic Surgery Training and Research Hospital, Kecioren, 06280 Ankara, Turkey; ^2^Department of Pathology, Ataturk Chest Disease and Thoracic Surgery Training and Research Hospital, Kecioren, 06280 Ankara, Turkey

## Abstract

Pulmonary endometriosis is a rarely seen disease of the lung. On computed tomography, a cavitary lesion of 15 × 26 in size was detected in the lung parenchyma of a 38-year-old female patient who was examined due to hemoptysis. The pathologic result of the surgically excised cavitary lesion was reported as pulmonary endometriosis.

## 1. Introduction

Endometriosis is the existence of endometrial tissue in the body areas out of the uterus. This pathology which is usually seen in the pelvis and abdomen may rarely affect lung parenchyma also [1]. Pulmonary endometriosis mostly clinically progresses in premenopausal women with catamenial symptoms such as hemoptysis, pneumothorax, or hemothorax, whereas asymptomatic cases are mostly observed as pulmonary nodules. Successful results may be achieved with hormonal therapy, but surgery can be considered in the cases with bronchoscopically defined hemorrhagic foci or the radiologically localized cases in the posttreatment recurrence.

## 2. Case Presentation

**Figure 1:**
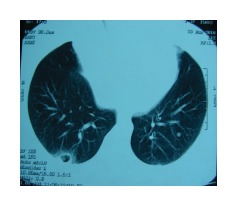
Computed chest tomography: thick-walled cavitary lesion of 15 × 26 mm in size in posterobasal segment of the right lung lower lobe.

**Figure 2:**
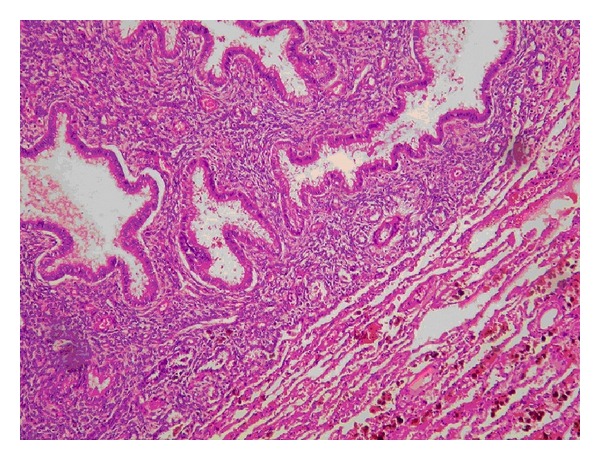
On pathology sections, lung parenchyma including focus of endometriosis containing stroma and glands and hemosiderin-laden macrophages in the adjacent area and hemorrhage sites (HE; ×200).

A 38-year-old female patient who was clinically followed up due to asthma for 4 years was evaluated for the complaint of blood expectoration existing for 3-4 months. It was learned from her history that the patient had hemoptysis that begins with menstruation, lasts 3 or 4 days, and ends spontaneously. Physical examination, routine laboratory outcomes, and bleeding parameters of the patient were in normal ranges. The patient learned not to have symptoms out of the menstruation period. On computed chest tomography, a thick-walled cavitary lesion of 15 × 26 mm in size was detected in the posterior segment of the right lung lower lobe (Figure 1). No pathology was observed in the bronchoscopy. The cavitary lesion was excised through video-assisted thoracoscopic surgery. The result of the pathology was reported as pulmonary endometriosis (Figure 2). There was not any problem in the 2-year followup of the patient who was not administered hormonal therapy.

## 3. Discussion

Diagnosis of pulmonary endometriosis is established with recurrent hemoptysis related to the menstruation and persistence of endometriosis foci demonstrated pathologically [1]. Thoracic endometriosis is generally seen as pleural involvement. Parenchymal pulmonary endometriosis is much more infrequent. It was described first by Schwarz in 1938 [1]. Occurrence mechanism of pulmonary endometriosis has not been fully clarified. Pleural implants might be explained by the cell islets migrating from the sinuses into the thorax and coelomic metaplasia, although the same explanation is not valid for pulmonary endometriosis [2].

Pulmonary endometriosis is radiologically observed in the form of pulmonary nodules or the clinical picture of hemoptysis, pneumothorax, or hemothorax in menstruation periods. Endometriosis foci may be located in trachea, bronchi, lung parenchyma, pleura, and diaphragm. In anamnesis of many patients, there is previous obstetric or gynecological pathology. Parenchymal or pleural involvement is usually at the right side [1]. In their paper reviewing 11 patients diagnosed with pulmonary endometriosis, Tatiana and Grant found in histories of 5 patients pregnancy-related problems or gynecological problems encountered in the prepregnancy period [2]. The most significant symptoms in these patients were recurrent hemoptysis, pneumothorax, or radiologically detected radiopacities in the lung.

Diagnosis of parenchymal endometriosis is difficult to be established radiologically. In general, plain radiographs do not provide information on the parenchyma disease. Radiologic findings are usually nonspecific and nondiagnostic [3]. Besides infiltration appearance, pulmonary endometriosis foci may have an aspect of ground-glass opacities, well-contoured nodular lesion, and rarely cavitary lesion. Given the history of our patient, lesion detected on the chest tomography was concomitant with catamenial hemoptysis, suggesting endometriosis focus. Aspect of the lesion and diameter and feature of the lesions may vary on computed tomography, especially during the menstruation period. Even there may be a parenchymal aspect between the menstruation periods [3].

Diagnosis should be obtained with bronchoscopic examination, and brushing will be performed during hemoptysis attack. However, this is of limited value. Following the bronchoscopy carried out in 21 patients considered having pulmonary endometriosis, endometrial cells could be found only in 4 patients [4]. Biopsy should be performed immediately before the onset of menses. Diagnosis may not be set when the biopsy is done during the menstruation [4].

In the differential diagnosis, the other causes of hemoptysis such as tuberculosis, pneumonia, bronchiectasia, tumor, and Goodpasture's syndrome must be ruled out first.

Medical and surgical treatment options can be considered for the treatment of pulmonary endometriosis disease. Most patients are free of problems during the medical therapy, but the complaints may begin again after the therapy is discontinued. Medical therapy should be primarily considered in case of the intrathoracic involvement. Danazol and Gonadotropin releasing hormon (GnRH) are medications of choice in this form of the therapy [1]. However, hormonal therapy is expensive, and the symptoms maybe repeated when the therapy is ceased. In addition, these drugs have side effects like menopausal symptoms [1]. Surgical treatment option should be considered in cases of the medical therapy being inadequate or ineffective. We first consider surgical treatment in our patient since there was a single pulmonary focus that might cause hemoptysis. Parenchymal lesion should be excised with thoracoscopic surgery if possible [5]. Surgical treatment requires long followup durations in the patients. However, it should be discussed whether hormonal therapy will be needed during the followup.
